# From Sensors to Data Intelligence: Leveraging IoT, Cloud, and Edge Computing with AI

**DOI:** 10.3390/s25061763

**Published:** 2025-03-12

**Authors:** Ilenia Ficili, Maurizio Giacobbe, Giuseppe Tricomi, Antonio Puliafito

**Affiliations:** 1 Department of Engineering, University of Messina, 98166 Messina, Italy; mgiacobbe@unime.it (M.G.); gtricomi@unime.it (G.T.); apuliafito@unime.it (A.P.); 2 Institute for High-Performance Computing and Networking of National Research Council of Italy (ICAR-CNR), 80131 Napoli, Italy; 3 National Interuniversity Consortium for Informatics, 00185 Rome, Italy

**Keywords:** edge computing, cloud computing, sensors, IoT, AI, federated learning

## Abstract

The exponential growth of connected devices and sensor networks has revolutionized data collection and monitoring across industries, from healthcare to smart cities. However, the true value of these systems lies not merely in gathering data but in transforming it into actionable intelligence. The integration of IoT, cloud computing, edge computing, and AI offers a robust pathway to achieve this transformation, enabling real-time decision-making and predictive insights. This paper explores innovative approaches to combine these technologies, emphasizing their role in enabling real-time decision-making, predictive analytics, and low-latency data processing. This work analyzes several integration approaches among IoT, cloud/edge computing, and AI through examples and applications, highlighting challenges and approaches to seamlessly integrate these techniques to achieve pervasive environmental intelligence. The findings contribute to advancing pervasive environmental intelligence, offering a roadmap for building smarter, more sustainable infrastructure.

## 1. Introduction

Moving into an era defined by hyper-connectivity and pervasive intelligence, the volume of data generated by sensors connected through the Internet of Things (IoT) has reached unprecedented levels. These data, originating from a wide range of applications spanning from smart homes to Industry 4.0, represent an invaluable resource for technological advancement. However, the real challenge lies in the ability to transform these raw data into useful information and, ultimately, actionable intelligence. The evolution of cloud computing and edge computing technologies today offers a powerful platform to address this challenge. Cloud computing provides scalable infrastructure for data processing and storage, while edge computing brings computation closer to the devices themselves, reducing latency and improving efficiency. When these technologies integrate with artificial intelligence (AI), new possibilities emerge for creating innovative solutions capable of learning, adapting, and responding in real time to user needs. This editorial explores how the convergence of IoT, cloud computing, edge computing, and AI is transforming the technological landscape, enabling new forms of distributed intelligence. Through an analysis of emerging trends and the latest developments, we aim to provide an overview of the opportunities and challenges within this fascinating technological ecosystem.

### 1.1. IoT and Sensor Networks: The Foundation of Real-Time Data Acquisition

The advent of the IoT [1] has radically transformed how data are collected and utilized across various sectors. At the heart of IoT are sensor networks, which form the foundation for real-time data acquisition. These networks consist of interconnected devices that monitor environmental, physical, and even biological parameters, producing a continuous stream of valuable data. With the proliferation of connected devices, IoT has enabled many applications, from smart homes and healthcare systems to industrial automation and urban management. One of the most significant advancements in IoT has been the evolution of smart sensors. These devices are no longer passive data collectors; they now have built-in processing capabilities, often directly incorporating AI or machine learning (ML) algorithms on the sensor. This on-device processing reduces the need for extensive data transmission to remote servers, thus decreasing latency and conserving bandwidth. For instance, smart temperature sensors in industrial settings analyze temperature variations locally. They can detect potential issues before they escalate into significant failures. This all happens without sending data to the cloud for initial analysis.

Another breakthrough in IoT is the development of sophisticated communication protocols that ensure efficient data transmission. As IoT networks grow, the demand for faster and more reliable communication has led to adopting technologies such as 5G, which provides ultra-low latency and high bandwidth capabilities. These advancements are crucial for applications like autonomous vehicles, where real-time communication between vehicles, infrastructure, and the cloud is essential for safety and decision-making.

Additionally, IoT systems increasingly rely on specialized networks like Low-Power Wide-Area Networks (LPWANs), which are designed for long-range communications with minimal energy consumption. LPWAN technologies like LoRaWAN have become vital in applications such as environmental monitoring, where sensors can operate over vast distances while requiring little power. Energy efficiency is another critical consideration for IoT and sensor networks, particularly for devices deployed in remote or difficult-to-access locations. The development of energy-harvesting technologies (such as solar-powered sensors or vibration-based energy systems) has extended the operational lifespan of these devices, reducing maintenance costs and the need for battery replacements. In harsh environments like offshore platforms or remote agricultural fields, these energy-efficient systems can function autonomously for years, providing continuous data without human intervention. Moreover, Li-ion batteries are important, especially for drones, due to their high energy density, long cycle life, and lightweight properties. Consequently, predicting their performance is crucial for enhancing drone flight safety, optimizing operations, and reducing costs [2].

The sheer scale of IoT sensor deployments introduces significant data management and integration challenges. The data streams generated by millions of IoT devices need to be processed, filtered, and stored to allow meaningful insights. Many IoT applications rely on edge computing, where processing is performed locally at or near the sensor, reducing the burden on centralized servers and enabling quicker decision-making. This distributed approach is crucial for minimizing delays in environments where real-time actions are required (such as in healthcare or industrial monitoring). However, as the number of connected devices increases [3], so does the complexity of managing these networks. Ensuring seamless connectivity and interoperability [4] between diverse devices and platforms remains a significant challenge. The industry is moving toward standardized communication protocols [5] and APIs that will make it easier to integrate various IoT systems, allowing for smoother data exchange and more cohesive ecosystems. The potential of IoT and sensor networks is vast, with applications spanning sectors as diverse as healthcare, agriculture, manufacturing, and environmental monitoring. In healthcare, IoT-enabled devices continuously track patient vitals, transmitting data to cloud platforms where AI systems can analyze them to predict health issues before they become critical. In agriculture, sensor networks monitor soil moisture and weather conditions, allowing farmers to optimize irrigation and increase crop yields while conserving water. In manufacturing, sensors embedded in machines can detect wear and tear, triggering maintenance requests before costly breakdowns occur. Despite the remarkable progress, the IoT landscape still faces several challenges. Security and privacy issues, mainly sensitive data transmitted across interconnected networks, remain a significant concern. Ensuring robust security frameworks, including data encryption and secure authentication, will be essential to building trust in IoT systems. Additionally, the increasing complexity of IoT networks presents scalability and management issues that must be addressed as the number of devices grows. IoT and sensor networks represent a transformative force, enabling real-time data collection and analysis across diverse domains. While significant advancements have been made, the continuous evolution of sensor technologies, communication protocols, and data management strategies remains crucial to unlocking their full potential. These networks are poised to redefine industries by driving efficiency, enhancing sustainability, and fostering innovation on an unprecedented scale.

### 1.2. Cloud-Based AI Architectures

Cloud computing [6] holds a pivotal role in the IoT ecosystem [7], serving as the computational backbone that enables the storage, processing, and analysis of the vast amounts of data generated by IoT devices. It transcends the limitations of local hardware and fragmented systems, unlocking the transformative potential of IoT and AI. One of the defining strengths of cloud computing lies in its scalability [8]. Cloud platforms dynamically adjust to the changing demands of IoT applications, managing surges in data traffic during events such as natural disasters or large-scale gatherings in smart cities. During periods of lower activity, resources can scale down, maintaining cost efficiency without sacrificing performance; this is a critical aspect for sustainable IoT deployments. Beyond scalability, the integration of AI into cloud platforms has redefined the way raw data is processed into actionable insights. Services such as AWS SageMaker, Google Vertex AI, and Microsoft Azure AI streamline the deployment of ML models. These platforms offer pre-trained models that can be tailored to specific applications, enabling real-time anomaly detection, predictive maintenance, and automated decision-making across various domains. In addressing the pressing concerns of data privacy and security, cloud providers implement advanced encryption techniques and granular access control mechanisms. These measures ensure the secure handling of sensitive data, such as healthcare records and financial information. Compliance with international regulations, including GDPR and HIPAA, further reinforces trust in cloud-based systems within industries where privacy is cardinal. The versatility of cloud computing extends to a wide range of applications. In healthcare, it facilitates telemedicine and remote patient monitoring, where real-time data inform critical medical decisions. In smart cities, it powers traffic optimization systems, reducing congestion and improving urban mobility. In industrial settings, cloud platforms act as hubs for predictive maintenance, enhancing operational efficiency and minimizing downtime. However, challenges remain. High bandwidth costs, latency issues, and the complexities of navigating data sovereignty laws pose ongoing difficulties. Solutions such as hybrid architectures that combine cloud and edge computing and renewable energy-powered data centers aim to address these hurdles. Cloud computing stands as a driving force in the evolution of IoT and AI ecosystems, paving the way for intelligent systems that are efficient, secure, and sustainable. By overcoming existing barriers and leveraging its strengths, the full potential of this technology can be realized.

### 1.3. Edge-Based Machine Learning

Edge computing [9] has emerged as a transformative approach in the IoT ecosystem, addressing the challenges associated with latency, bandwidth, and real-time decision-making. By processing data closer to their source (at the “edge” of the network), this paradigm reduces the dependence on centralized cloud systems. For latency-sensitive applications such as autonomous vehicles, industrial automation, and remote healthcare, edge computing is not merely an enhancement but a necessity [10]. One of the most impactful advancements in this field is the ability to perform AI inference directly on edge devices. Frameworks like TensorFlow [11] and PyTorch Mobile enable lightweight, optimized AI models to run on resource-constrained devices. This capability allows edge systems to analyze data locally, eliminating the need to send vast amounts of information to distant cloud servers. For example, in video surveillance systems, edge devices equipped with AI can detect anomalies or security threats in real time, significantly improving response times while conserving network bandwidth. Hybrid architectures that seamlessly integrate cloud and edge computing are further enhancing the versatility and efficiency of IoT ecosystems. These systems dynamically allocate workloads between cloud and edge environments based on factors such as processing power, data urgency, and network conditions. Critical, time-sensitive tasks are executed at the edge, while more resource-intensive computations, such as training deep learning models, are relegated to the cloud. This division optimizes resource use and ensures that applications maintain high performance, even under varying conditions. The applications of edge computing [12] span a wide range of industries. In healthcare, wearable devices equipped with edge capabilities can monitor vital signs and alert medical professionals to irregularities in real time. In manufacturing, edge-based systems power predictive maintenance by identifying equipment issues before they lead to costly breakdowns. Similarly, in smart cities, edge computing supports traffic management systems that adapt dynamically to real-time conditions, reducing congestion and improving urban mobility. Despite its many advantages, edge computing faces several challenges. Resource constraints on edge devices, such as limited power and memory, often require trade-offs between performance and functionality. Additionally, ensuring security in decentralized architectures is complex, as each edge device becomes a potential attack surface. Addressing these challenges involves innovations such as lightweight encryption methods, decentralized trust frameworks, and energy-aware computing algorithms tailored for edge environments.

### 1.4. Edge AI vs. Cloud AI

AI serves as the critical bridge between raw data and actionable insights, empowering systems to make decisions, predict outcomes, and automate processes with unprecedented accuracy and efficiency. In the context of IoT, cloud, and edge computing, AI is the force that transforms vast, unstructured data streams from sensors into meaningful, context-aware intelligence. One of the primary roles of AI is in processing unstructured sensor data. The data collected from IoT devices often include diverse formats such as text, images, audio, and time-series signals. AI techniques, including ML [13] and deep learning [14], excel at identifying patterns, correlations, and anomalies within these heterogeneous datasets. For instance, in environmental monitoring, AI algorithms can analyze complex combinations of temperature, humidity, and air quality readings to predict weather patterns or detect pollution spikes, offering actionable insights to policymakers. Predictive analytics [15] is another domain where AI has made significant strides. By training predictive models on historical and real-time data, AI can forecast potential failures and recommend preemptive actions. In industrial environments, for example, sensors embedded in machinery generate continuous data streams on vibrations, temperature, and pressure. AI systems can analyze these streams to predict when a machine component is likely to fail, allowing for timely maintenance and minimizing downtime. This predictive capability is equally transformative in other fields, such as healthcare, where AI-powered systems can anticipate medical emergencies based on the continuous monitoring of patient vitals. AI also enables automation in time-critical scenarios where rapid decision-making is essential. Autonomous vehicles, for instance, rely on AI to process data from cameras, LiDAR, and radar in real time, identifying obstacles and making split-second navigation decisions. Similarly, in disaster-response applications, AI systems deployed at the edge can analyze real-time data from drones and sensors to prioritize rescue operations and allocate resources effectively. However, the integration of AI into IoT systems also introduces challenges. Developing reliable AI models requires access to large, high-quality datasets, which can be difficult to obtain in some domains. Furthermore, the computational demands of AI algorithms can strain resource-constrained devices, particularly at the edge. Addressing these limitations has led to the emergence of innovative techniques such as federated learning, where models are trained collaboratively across decentralized devices without sharing raw data, ensuring privacy and reducing computational overhead.

## 2. Edge and Cloud: Complementary Pillars for AI-Driven IoT

Ref. [16] shows how the convergence of IoT, cloud, edge computing, and AI has unlocked a huge potential. However, this scenario also presents a complex landscape of challenges that need to be addressed to fully realize the vision of interconnected intelligent systems. These challenges span technical, operational, and security domains, reflecting the multifaceted nature of these technologies. Among the many challenges facing IoT ecosystems, managing the vast amounts of data that they generate stands out as a particularly complex issue. With billions of connected devices continuously collecting and transmitting information, the scale of data produced has reached once unimaginable levels. These data are not only immense in volume but also highly dynamic, flowing at rapid speeds and encompassing a wide variety of formats, from numerical readings to audio and video streams.

Handling such data across distributed systems requires sophisticated strategies to address challenges related to storage, processing, and integration. As the number of devices and sensors in a network grows, so does the difficulty in ensuring that the data remain accurate, reliable, and consistent. Without careful management, issues such as data duplication, inconsistency, or loss can undermine the effectiveness of IoT applications. To navigate these complexities, robust data governance frameworks are becoming indispensable. These frameworks provide the policies and tools needed to maintain data integrity and ensure compliance with privacy regulations. Another pressing challenge in IoT ecosystems revolves around the limitations of edge devices, which are fundamental to the IoT and edge computing paradigm. These devices often operate under strict constraints in computational power, memory, and energy availability. Unlike cloud servers with virtually unlimited resources, edge devices are tasked with performing complex computations on limited hardware. This scenario demands innovative approaches to optimize resource utilization without compromising performance. Solutions such as lightweight AI models, energy-efficient hardware, and intelligent task allocation that strategically distributes workloads between cloud and edge environments have emerged as crucial strategies for addressing these limitations. In addition to resource constraints, security, and privacy remain ongoing concerns. As data flow across IoT devices, cloud infrastructures, and edge nodes, the risk of breaches, unauthorized access, and data manipulation grows. The decentralized nature of these networks, combined with the sensitive nature of the data that they handle, amplifies the urgency of robust security mechanisms. Traditional approaches to cybersecurity are proving inadequate in the face of evolving threats. To mitigate these risks, advanced measures such as end-to-end encryption, secure boot processes, and real-time threat detection have become indispensable. These tools not only safeguard sensitive information but also reinforce trust in IoT deployments [17]. In a real scenario, such as the application of intelligent video-surveillance systems in smart cities to ensure security and the monitoring of traffic or crowds, managing and protecting video streams is a challenge. Although combining edge computing and machine learning techniques [18] allows one to transmit data in textual form, avoiding the transmission of videos or images, this is not always applicable due to technological or environmental limits. In this case, managing and protecting video streams poses a challenge, especially when transmitting and storing sensitive data in the cloud. An example concerns today’s networking infrastructures such as Kubernetes, in which scalability has increased the demand for everything-as-a-service concepts, including encryption services. In [19], the authors detail the deployment of an Encryption as a Service (EaaS) framework on Kubernetes. Integrating Django, PostgreSQL, and Nginx into Docker containers managed by Kubernetes has shown promising results, especially in handling HTTP/HTTPS traffic and interservice communications.

### 2.1. Challenges in Deploying Edge Computing at Scale

Deploying processing models at the edge, particularly for AI applications, introduces several critical challenges. While model implementation can be abstracted from hardware, edge devices still face significant limitations in computational power, memory, and energy availability. Overcoming these constraints while ensuring cost efficiency and consistent performance across heterogeneous environments requires innovative solutions, such as modular hardware and edge-specific design. Network reliability is another key challenge, especially in scenarios with intermittent connectivity or fluctuating network performance. Techniques like adaptive caching, delay-tolerant networking, and edge–cloud collaboration are crucial in mitigating these issues, ensuring reliable operation even under challenging conditions. Maintaining data consistency across distributed edge devices presents additional complexity. Since edge systems often operate asynchronously, reconciling updates across a distributed architecture is essential. Advanced distributed consensus algorithms and conflict-free replication mechanisms are critical to achieving consistency without compromising performance. Addressing these interconnected challenges is essential for unlocking the full potential of edge computing in large-scale deployments.

### 2.2. Ethical Challenges in Data-Driven Decision-Making

Biased datasets or poorly designed algorithms can perpetuate or even amplify existing inequalities. Algorithmic bias, therefore, is one of the most critical issues. For example, facial recognition systems have been shown to exhibit lower accuracy rates for underrepresented demographic groups, thus potentially leading to discriminatory outcomes. Addressing this issue requires not only more diverse datasets but also transparent and explainable AI systems that allow for scrutiny and accountability.

Data privacy is another issue in light of the previously unheard-of volumes of data collection. Without robust safeguards and the adoption of secure mechanisms, the combination of IoT and edge computing can be misused for intrusive monitoring, violating privacy rights, and eroding public trust. As a consequence, strong legal frameworks and privacy-preserving technologies, such as federated learning and differential privacy, are crucial to reducing these risks. The traditional centralized architecture of federated learning suffers from several problems, e.g., a single point of failure and communication bottlenecks, especially malicious servers that analyze gradients to infer sensitive information, leading to gradient leakage. To address these issues, a robust and privacy-preserving decentralized federated learning (RPDFL) scheme is presented and discussed in [20]. Experimental results show that RPDFL is significantly superior to standard federated learning methods in terms of model accuracy and convergence and is suitable for digital healthcare applications. Practical use cases of federated learning for medical diagnostic tools are discussed in [21]. Federated learning enables collaborative model training on decentralized data, balancing improved healthcare insights with privacy and security. The result is an optimization in therapeutic outcomes, also ensuring the security and confidentiality of patient data in alignment with regulatory standards.

Finally, accountability, fairness, and autonomy are fundamental ethical considerations in data-driven decision-making. Autonomous computation generates insights that directly impact people’s lives, from credit approvals to medical diagnoses. Therefore, future data-driven ecosystems must incorporate ethical principles into system design to foster trust and uphold ethical standards.

### 2.3. The Edge as the Front Line in Inference and Pre-Processing

In the realm of ML, edge computing plays a crucial role in executing inference. When an IoT device collects real-time data, edge computing enables the application of pre-trained models directly on local devices to provide quick responses. For instance, in industrial environments, vibration sensors equipped with local pre-trained models (such as Support Vector Machines (SVMs) or Convolutional Neural Networks (CNNs)) can detect anomalies that signal potential equipment failures. The speed of this inference is vital for timely process interruption and preventing damage, such as the overheating of a machine or the failure of a component. In the context of deep learning, edge computing proves invaluable for executing computationally intensive inference tasks, such as those required by Deep Neural Networks (DNNs), on site. However, these deep networks require significant computational resources, often exceeding the capabilities of traditional edge devices. To address this challenge, techniques like quantization and pruning are frequently applied to models, reducing computational complexity and adapting DL models for execution in edge environments with limited resources. Recent research investigates model compression strategies to overcome resource restrictions in edge devices used for deep learning applications. Global pruning, knowledge distillation (KD), and quantization are promising methods to reduce model size and improve efficiency [22]. KD has been shown to be particularly successful in reducing model size with minimum accuracy loss, whereas 8-bit quantization, while efficient, frequently results in accuracy erosion. Furthermore, a two-stage compression technique combining structured channel pruning and quantization has been found to effectively reduce both model size and computing demands in edge-based activity identification tasks [23]. These strategies allow deep learning models to perform well within the limits of edge computing settings. An exemplary implementation of edge and cloud synergy is provided by the Distributed Incremental Learning over Cloud and Edge Computing (DILoCC) framework [24]. DILoCC leverages the computational power of the cloud and the proximity benefits of edge computing to optimize ML workflows. At the edge, the system deploys Lightning Rod, an agent running on Linux-based devices that manages IoT endpoints and facilitates the deployment of plug-ins for data collection and pre-processing. For example, Lightning Rod can collect real-time sensor data, organize it into training batches, and perform incremental training or inference locally using lightweight models.

**Figure 1:**
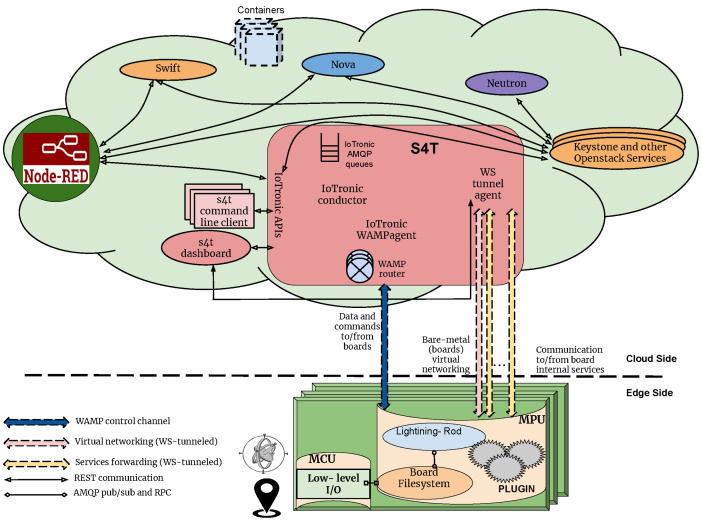
DILoCC general architecture.

This approach minimizes latency and ensures that critical tasks are performed close to the data source. Meanwhile, the cloud acts as an orchestrator, providing lifecycle management for edge devices and hosting resource-intensive operations. Notably, Node-RED, as indicated in Figure 1, plays a role in facilitating edge–cloud interactions through its flow-based programming model, enabling seamless data processing and integration. By strategically distributing tasks across edge and cloud resources, DILoCC addresses challenges such as latency, resource limitations, and scalability. The architecture demonstrates how integrating edge devices with a robust cloud backend can improve ML workflows in dynamic environments, such as predictive maintenance in industrial environments or adaptive learning systems in IoT networks.

**Figure 2:**
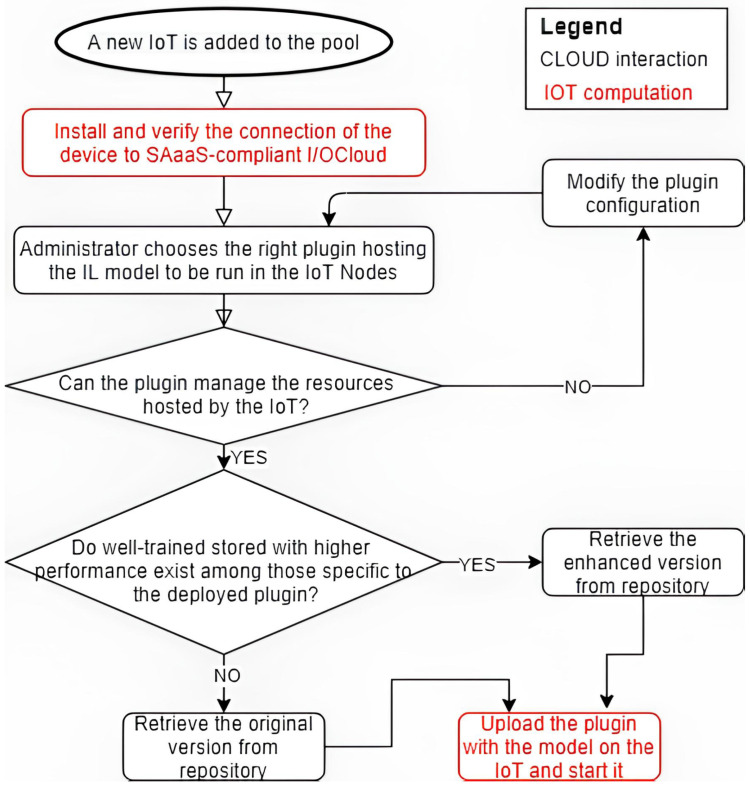
Device enrollment process.

**Figure 3:**
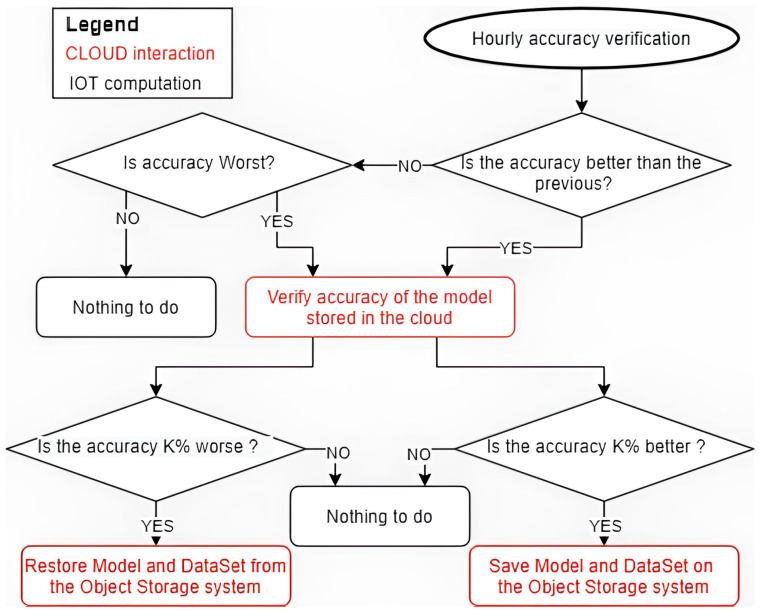
Self-monitoring procedure.

A further clarification of the synergy between edge and cloud introduced by DILoCC follows two of the main workflows put in place to manage the solution: the “device enrollment” and “self monitoring procedure”, as can be shown in Figure 2 and Figure 3.

The device enrollment process consists of the following key steps:

- Preparing the IoT Device: The first step involves setting up the IoT device. This process is managed by a device-centric sensing system running in the cloud, specifically using Stack4Things (S4T), an unofficial project within the OpenStack ecosystem that serves as a reference implementation for the I/OCloud paradigm. During this phase, the Lightning Rod component is installed and configured to establish a connection with the system. This step represents an interaction between Node-RED and Stack4Things (S4T). Additionally, the device is registered and activated, and its hosted services are made available;
- Injecting the Incremental Learning (IL) Model: Once the device is connected, the administrator can deploy a compatible Incremental Learning (IL) model based on the device’s physical configuration;
- Model Adaptation: As described in the system architecture, the IL model receives input from IoT-managed devices (sensors and actuators). This implies that, in some cases, modifications to the model are required to ensure proper integration;
- Model Enhancement and Deployment: Before the model and its dataset are deployed onto the device, the system verifies whether an enhanced version of the model is available. This is possible because each model has an incrementally trained version, generated through continuous learning on IoT devices. These versions are stored in the cloud and linked to the original template model using a UUID. This UUID enables tracking improvements by associating the model version with the accuracy achieved during testing.

The system enables multiple checks; for performance-monitoring procedures; the most relevant is performed by the devices themselves. This procedure is described synthetically by the follows steps:

- Regular Accuracy Verification: A plugin injected into the IoT devices performs an accuracy check for every fixed slot of time. After each training phase, a test phase follows, where the model evaluates its accuracy and compares it with the previously recorded accuracy;
- Model Accuracy Comparison: If the model’s accuracy has changed, the plugin compares its current accuracy with the enhanced version stored in the cloud repository. The system checks whether the accuracy retrieved from the cloud, with a timing defined by a fixed threshold (set in the plugin during injection), is higher than the current model accuracy;
- Recovery Phase (Model Update from Cloud): If the cloud version performs better than the device’s model (by at least the threshold), the system initiates a recovery phase. This forces the cloud to inject a better model and its corresponding training batch onto the IoT device. The training batch is deleted once the training process is complete;
- Cloud Model Update (Snapshot Saving): Conversely, if the IoT device’s model outperforms the cloud’s stored version (by at least the threshold), the plugin forces the cloud to save the current model and dataset as a new snapshot in the repository.

### 2.4. The Cloud for Training and Managing Complex Models

While edge computing is crucial for real-time inference, training ML and deep learning models requires significantly more computational power. Deep Neural Networks (DNNs), such as transformer architectures or Recurrent Neural Networks (RNNs), require vast amounts of data and computational resources, making cloud computing indispensable for their training. The cloud allows for distributing and parallelizing the training of models across high-performance Graphic Processing Unit (GPU) or Tensor Processing Unit (TPU) clusters, significantly accelerating training times. This is essential, for example, in computer vision, where complex DL models must be trained on large labeled datasets from cameras and IoT sensors. In addition to training, the cloud also offers a powerful platform for continuous model updates. In dynamic IoT environments, where usage conditions can change rapidly, it is crucial to update ML and DL models to maintain inference accuracy. The cloud enables the collection of data from various edge devices and the retraining of models, improving them with new information. Once updated, the model can be synchronized with edge devices, ensuring that local inferences are always based on optimized and up-to-date algorithms.

In such a context, cloud-based frameworks play a pivotal role in providing the necessary computational power and flexibility for these tasks. By extending traditional cloud management systems into the realm of IoT, these frameworks enable the seamless integration of diverse resources, facilitating the development and deployment of sophisticated ML and data analysis models. One such solution is Stack4Things (S4T) [25], an open-source research project and innovative framework developed by the University of Messina. The S4T architecture is composed of a cloud-side component, IoTronic, and one or more edge-side components called Lightning Rod (LR). These elements work together to enable efficient IoT device orchestration, remote management, and real-time data processing, bridging the gap between cloud and edge computing.

**Figure 4:**
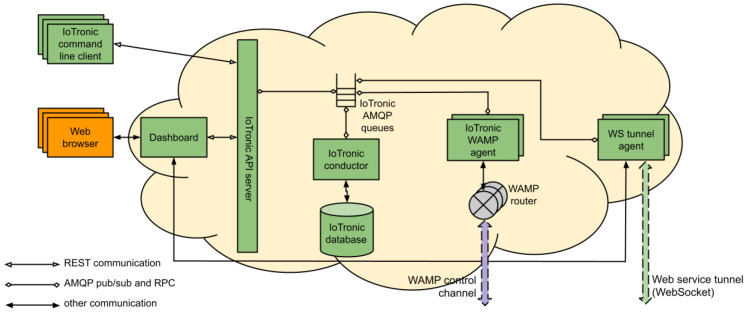
IoTronic’s architectural schema.

As illustrated in Figure 4, IoTronic engages with the LR device-side agent to establish and sustain a robust connection between the cloud and edge devices, effectively overcoming challenges posed by network address translations (NATs) and stringent firewalls. This seamless connectivity is powered by WebSocket technology, leveraging the Web Application Messaging Protocol (WAMP) to enable a full-duplex messaging channel, ensuring efficient and reliable communication.

S4T bridges the gap between OpenStack’s cloud capabilities and the requirements of IoT environments. The Input/Output (I/O) cloud approach [26] further enhances this by leveraging S4T’s functionalities to provide standardized, generic programming interfaces for IoT resources, regardless of the underlying infrastructure configuration.

In [27], the authors investigate and develop energy-aware architectural models and edge/cloud computing technologies to design next-generation, deep-learning-enhanced, and self-conscious IoT-extended Distributed Cyber-Physical Systems (DCPSs). The proposed solution was validated through case studies on optimizing renewable energy communities (RECs). RECs consist of individuals, organizations, and businesses collaborating to produce and consume renewable energy, such as solar or wind power. Specifically, RECs comprise multiple real estate units, sufficient to justify a dedicated production setup. Each unit is equipped with a general smart meter, and its electrical systems are intentionally divided into multiple subsections connected to specific sub-meters. This design enables granular energy consumption monitoring and management. Smart meters are integrated with IoT devices such as Raspberry Pi 3, Arancino [28], and other CPU- and MCU-based edge devices, capable of managing the LR edge component of S4T. Deep learning (DL) models are deployed as plug-ins injected into these edge devices through S4T, enabling advanced data-driven decision-making.

For the use case, two different public datasets have been used: (i) The household electricity load diagrams 2011–2014 dataset, designed by the University of California, School of Information and Computer Science, and shared in the UCI ML repository [29], and (ii) The solar power generation dataset [30]. Moreover, two types of DL models are developed: long short-term memory (LSTM) and bidirectional long short-term memory (BiLSTM) neural network-based models, useful for processing sequential data and capable of learning long-term dependencies [31]. The LSTM and BiLSTM models were trained on production and consumption datasets, respectively. More specifically, LSTM was trained to predict both the DC power and efficiency of an inverter, while the BiLSTM was trained to predict Global Active Power (GAP) by using all other consumption features.

**Figure 5:**
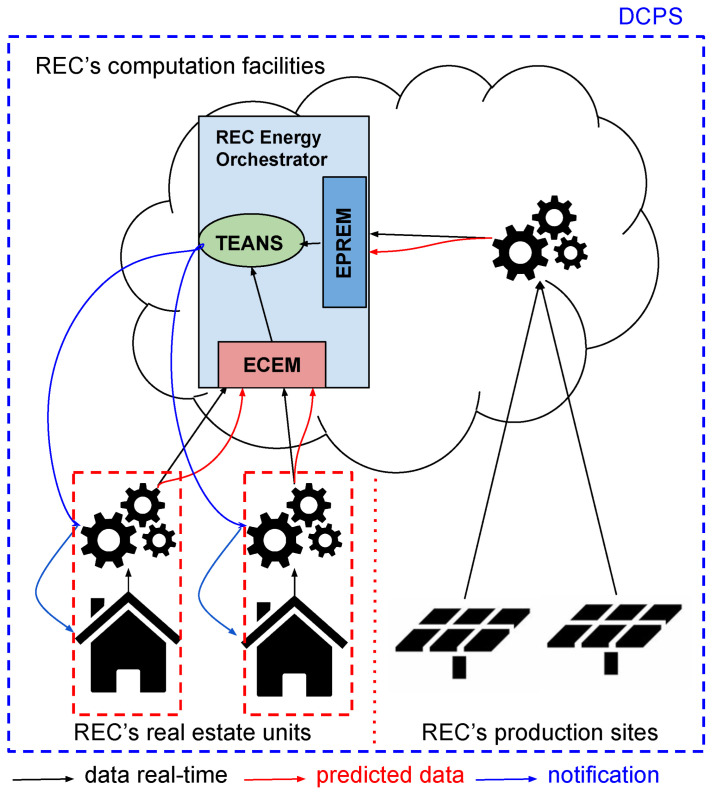
REC architecture. ECEM stands for Energy Consumption Estimation Module, EPREM stands for Energy Production Estimation Module, and TEANS stands for Threshold Evaluator and Notification System.

**Figure 6:**
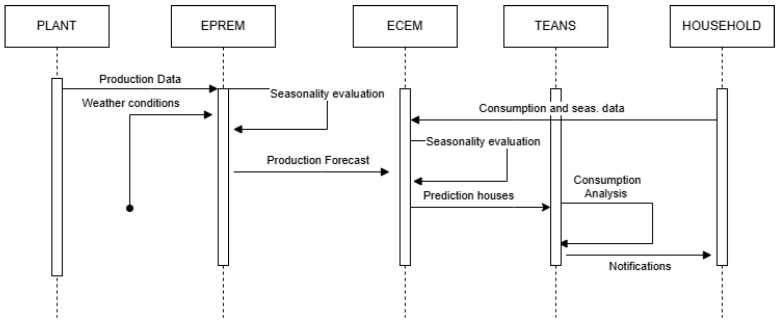
Sequence diagram analysis.

As illustrated in Figure 5, the energy orchestrator at the cloud side of the REC consists of three primary modules. The first two, the Energy Consumption Estimation Module (ECEM) and the Energy Production Estimation Module (EPREM), are designed to analyze both real-time and predicted data to approximate energy consumption and production values. The third module, identified as the Threshold Evaluator and Notification System (TEANS), leverages these approximations to determine whether energy demands exceed the community’s available resources. If such a discrepancy is identified, TEANS seeks to implement mitigation strategies—for instance, in the case of unexpected consumption spikes, notifications may be sent to community members with higher usage patterns. To provide further clarification of the described process, the workflow is illustrated more comprehensively in the accompanying figure using a sequence diagram (Figure 6).

The orchestrator implemented within the DCPS has demonstrated its effectiveness in sending notifications to users based on data collected and processed by AI models, helping them to make informed decisions. By leveraging AI for short-term forecasting, it provides accurate predictions of energy consumption and production. These forecasts are instrumental in optimizing energy usage within RECs, ensuring that community resources are allocated efficiently and disruptions due to unexpected demand are minimized. The integration of advanced deep learning techniques, such as LSTM and BiLSTM models, enables the orchestrator to achieve a high level of accuracy and reliability in its estimations.

### 2.5. Hybrid Architectures: Training in the Cloud and Inference at the Edge

**Figure 7:**
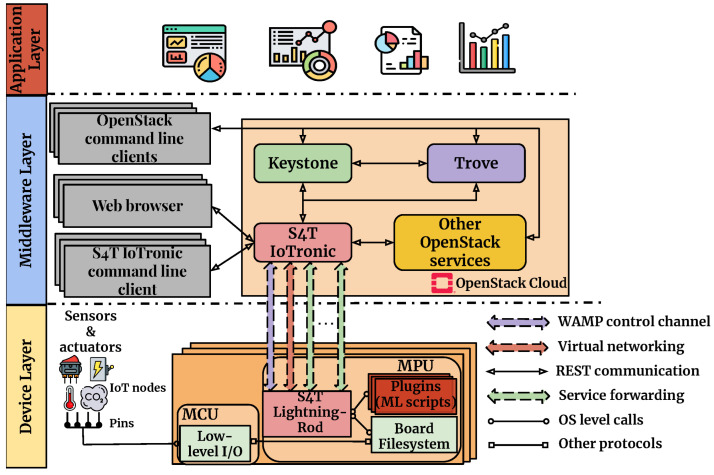
The SHIRS architecture.

One of the most powerful architectures in the synergy between edge and cloud is the *hybrid cloud–edge* paradigm, which combines centralized training in the cloud with distributed inference at the edge. This architecture leverages the computational power of the cloud to train sophisticated and optimized models, which are then deployed on edge devices for real-time execution. This distributed deployment model is ideal for applications such as the real-time monitoring of autonomous vehicles or drones, where quick local decisions need to be made based on complex deep learning models. For example, in intelligent video surveillance applications, facial recognition or behavior analysis models can be trained on large datasets in the cloud, using advanced techniques such as Convolutional Neural Networks (CNNs) or transfer learning architectures to improve performance in complex environments. Once trained, an optimized version of these models can be deployed on edge devices (such as smart cameras), which perform real-time inference, detecting and identifying suspicious behaviors and immediately alerting law enforcement or security systems. This hybrid approach is exemplified by the SHIRS architecture, shown in Figure 7, for indoor air quality (IAQ) monitoring through a multi-layered architecture [32]. The proposed SHIRS system has been tested in a university setting, specifically in a multifunctional room used for meetings, laboratory work, seminars, and presentations. This real-world case study highlights the system’s ability to monitor and manage indoor air quality effectively, ensuring a healthy and comfortable environment for diverse academic and professional activities. At the device layer, edge devices equipped with microprocessors (MPUs) process the raw sensor data locally, enabling real-time responses such as the activation of ventilation systems when pollutants such as CO_2_ or CO exceed threshold values. Connecting these devices to the cloud for secure management and advanced analytics is the middleware layer built on Stack4Things. Pre-processed data are sent to the cloud, where ML models, trained on historical data, improve the accuracy of the predictions. Finally, at the application layer, SHIRS enables real-time applications such as HVAC optimization and health alerts.

By combining edge inference with periodic cloud updates, SHIRS ensures responsive and efficient air quality management, ideal for environments like schools or workplaces.

## 3. Fundamental Technological Pillars

### 3.1. Seamless IoT–Cloud–Edge–AI Integration

Seamless IoT–cloud–edge-AI integration refers to the coordinated interaction of IoT devices, edge computing, cloud platforms, and AI technologies to enable efficient, scalable, and intelligent systems [33]. Future advancements in this domain will focus on developing interoperable protocols [34] (e.g., MQTT, CoAP) and standardized data formats to ensure seamless communication across heterogeneous devices and platforms. Edge computing will play a key role in low-latency, real-time data processing, while the cloud will provide scalable AI model training and data storage. AI-driven decision-making will be decentralized, allowing for edge-based inference and cloud-based model updates, which will enhance system responsiveness and efficiency.

Efficient ML and deep learning model lifecycle management is crucial to ensuring reliability in IoT systems [35]. The cloud provides a platform to monitor and optimize the performance of distributed models, enabling regular updates and the continuous improvement of models through techniques such as federated learning or continual learning. Federated learning [36], for example, allows models to be trained directly on edge devices without the data ever leaving the device, preserving privacy and improving the scalability of operations. Models are updated centrally in the cloud, while inferences remain local, reducing security risks and enhancing privacy.

Key to this seamless integration is the use of resource orchestration frameworks like Kubernetes [37] to manage workloads across distributed environments, ensuring optimal resource allocation. As IoT networks expand, future systems will need to support dynamic scalability, adapting in real time to varying data volumes and computational demands. Furthermore, security and privacy will remain paramount, with integrated solutions focusing on end-to-end encryption, secure data transmission, and AI model protection to safeguard sensitive information across the entire IoT–edge–cloud–AI pipeline. This integrated approach will pave the way for more intelligent, responsive, and sustainable applications in industries such as healthcare, manufacturing, and smart cities.

### 3.2. Real-Time Intelligence

The Seamless IoT–cloud–edge-AI integration is closely linked to the concept of Real-Time Intelligence, as it enables the immediate and intelligent processing of data generated by IoT devices, leveraging the power of edge computing and cloud to make real-time decisions. The ability to seamlessly integrate AI between IoT, edge, and cloud allows for inferences to be made directly on IoT devices or near the data source (edge), reducing latency and improving system responsiveness. Additionally, processing large amounts of data in the cloud allows for training complex AI models, which are then deployed to edge devices for fast, context-aware decision-making. This continuous flow of data and real-time decisions is critical in scenarios like smart cities [38], healthcare [39,40], and predictive maintenance [41], where the timeliness and accuracy of information can enhance operational efficiency and safety. The integration between IoT, edge, and cloud is achieved through a combination of decentralized architectures and intelligent coordination mechanisms. For instance, blockchain ensures secure and tamper-proof data exchange by maintaining a distributed ledger that eliminates single points of failure. Dynamic workload balancing algorithms monitor real-time system demands, redistributing computational tasks between edge and cloud environments to minimize delays and optimize throughput.

The ability to process data in real-time near the source reduces the need to transmit large volumes of data to the cloud, thereby lowering energy consumption and the environmental impact of communication networks.

### 3.3. Sustainability

Sustainability is an increasing priority in the IoT–cloud–edge domain, focusing on energy optimization and reducing the environmental impact of distributed technologies. In IoT systems, the adoption of low-power protocols, such as LoRaWAN [42] and Bluetooth Low Energy (BLE) [43], and the use of energy-efficient microcontrollers, such as those based on ARM Cortex-M, are essential for extending battery life and reducing overall consumption. These protocols achieve energy savings by reducing data transmission intervals and employing duty-cycling mechanisms that switch devices to low-power states when idle. Edge computing plays a critical role by processing data closer to the source, minimizing latency, and reducing the need to transmit large volumes of data to cloud data centers, thereby mitigating the energy impact of data transport networks. Technologies like Docker and Kubernetes, optimized for the edge (e.g., K3s), enable the efficient deployment of applications on resource-constrained devices. In the context of AI applications, the use of lightweight models, such as those developed with TensorFlow Lite [44] or PyTorch Mobile [45], allows for inference with minimal energy consumption. For training, the use of optimized hardware such as TPU or GPU with low power consumption, along with model compression techniques like pruning and quantization, significantly reduces computational impact. In data centers, the integration of renewable energy and the use of metrics like Power Usage Effectiveness (PUE) and Data Center Infrastructure Efficiency (DCIE) ensure the monitoring and improvement of energy efficiency [46]. Therefore, the future of AI and ML in energy management systems (EMSs) has potential, with the ability to develop EMS methods through innovative advancements [47]. Future directions include the development of AI-driven dynamic energy management systems that leverage predictive analytics to optimize resource allocation in real time. The adoption of distributed AI architectures, where training and inference are dynamically split between edge and cloud based on energy availability, represents a promising avenue for balancing performance and sustainability.

Table 1 summarizes open-source techniques and tools for achieving energy efficiency in deep learning for sustainable AI development, especially for applications on resource-constrained devices:

In the direction of energy optimization, another future perspective delves deeper into techniques that aim to enable devices to harvest energy from wireless communication. Works like [41,42] delve deeply into the investigation of wireless-powered multi-access edge computing (WP-MEC) to offload computational tasks that will be performed by exploiting the energy harvested previously. In [43], an approach to exploiting an ML model to use both the WP-MEC and IRC techniques at the same time is analyzed.

### 3.4. Standardization

Standardization in the IoT domain, particularly in edge/cloud systems, plays a crucial role in ensuring interoperability, scalability, and security in increasingly complex ecosystems. In the IoT domain, the standardization of communication protocols such as MQTT, CoAP, and Lightweight Machine to Machine (LwM2M) [48] ensures that heterogeneous devices can exchange data efficiently. When these data are processed at the edge, especially in AI applications, it is essential to adopt common standards for AI model deployment and resource orchestration, such as Kubernetes for containers or Open Neural Network Exchange (ONNX) for ML formats. Cloud platforms must support open APIs and interoperable languages to facilitate seamless integration with existing and future systems. Finally, ethical and regulatory standards [49], such as those related to privacy (GDPR) and the responsible use of AI [50,51], are fundamental to ensuring that the entire IoT–edge–cloud stack operates transparently, reliably, and securely. In the AI domain, ONNX [52] enables interoperability between ML frameworks, and TensorFlow Lite is often used for edge implementations. However, there are several alternatives to ONNX, including Neural Network Exchange Format (NNEF) [53], Portable Format for Analytics (PFA) [54], TensorFlow Extended (TFX) [55], etc. The choice of alternative depends on the ecosystem in which one is working and the specific goals of the project.

Regarding data formats and protocols, Open Platform Communications Unified Architecture (OPC UA) [56] ensures secure and standardized information exchange between industrial devices, while LoRa and the narrowband Internet of Things (NB-IoT) are common standards for long-range, low-power IoT communications; some comparisons are detailed in Table 2.

Finally, security and privacy guidelines, like those provided by ISO/IEC 27001 [57] are essential to ensure data protection and compliance with regulations across all levels of the IoT–edge–cloud architecture.

## 4. Bridging Vision and Reality: Enabling Technologies in Action

A practical demonstration of these enabling technologies can be observed in the IoT ecosystem implemented in the city of Caltanissetta [58]. This urban initiative showcases how an integrated approach, leveraging advanced communication protocols and data visualization platforms, can address complex urban challenges while fostering innovation and sustainability. The system implements specific vertical applications, i.e., ‘Edge Park Finder’, ‘Edge Traffic Flow’, and ‘Weather And Pollution Monitoring’ based on the 4rancino.ai^®^ technology [59].

At its core, the system relies on the S4T platform, utilizing Elastic–Kibana dashboards for efficient data management and visualization. These dashboards, hosted within the SmartMe cloud environment, enable users to analyze real-time data with precision, offering actionable insights into traffic conditions, parking availability, and energy consumption. The intuitive design of the dashboards facilitates trend identification, anomaly detection, and the customization of visualizations to meet diverse user needs.

**Figure 8:**
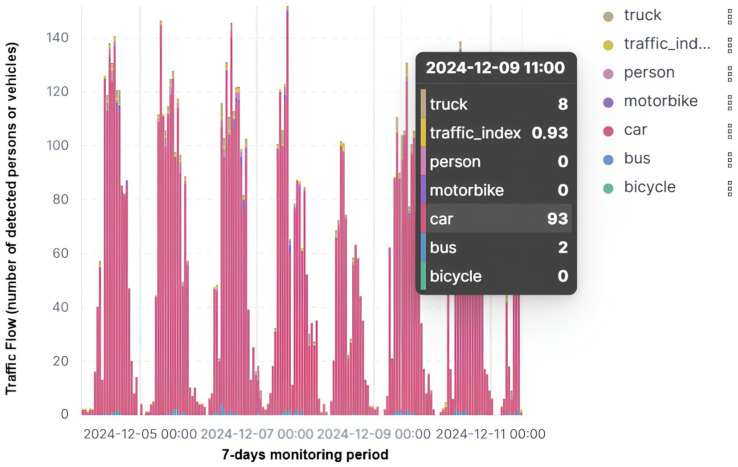
Seven-day monitored traffic flow in the city center of Caltanissetta.

Figure 8 shows a screenshot of the Elastic–Kibana dashboard implemented for the municipality of Caltanissetta, concerning a weekly monitored traffic flow in its urban center through the ‘Edge Traffic Flow’ vertical application. Specifically, the allowed user can visualize the number of detected persons or vehicles both in numerical and in graphical manner.

**Figure 9:**
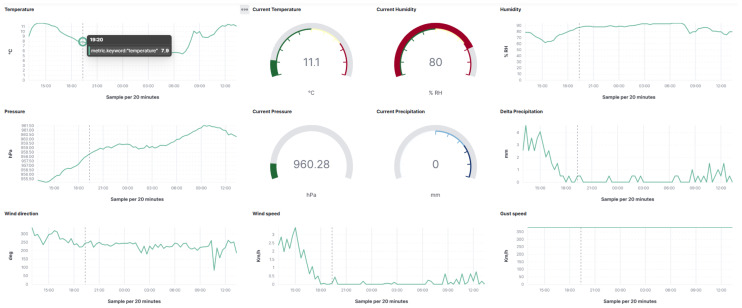
Twenty-four-hour monitored weather parameters in Caltanissetta.

Figure 9 shows a screenshot of the Elastic–Kibana dashboard implemented for the municipality of Caltanissetta, concerning daily monitored weather conditions in its urban center through the ‘Weather And Pollution Monitoring’ vertical application.

At its core, the ecosystem relies on Cyber-Physical Systems (CPSs) strategically distributed throughout the city, forming a connected network that spans various urban applications. These systems utilize LoRaWAN, a wireless communication protocol designed specifically for IoT, to achieve long-range and low-power communication. LoRaWAN operates in the unlicensed ISM (Industrial, Scientific, and Medical) bands and has official regional specifications, called Regional Parameters, that are possible to download from the LoRa Alliance website. Among these, EU868 (863–870 MHz) is used in Europe, US915 (902–928 MHz) in U.S.A., AS923 (923 MHz) in parts of Asia, KR920 (920–923 MHz) in South Korea, CN470 (470–510 MHz) in China, AU915 (915–928 MHz) in Australia, IN865 (865–867 MHz) in India, and RU864 (864–870 MHz) in Russia. Local regulations must be followed for each country. This capability is essential for enabling cost-effective and energy-efficient data transmission across the urban landscape.

This dual communication framework creates a robust and scalable infrastructure. LoRaWAN provides the foundation for widespread connectivity, ideal for low-bandwidth applications, while LTE/4G enables high-speed data transmission from the gateway to the cloud. The synergy between the two technologies also ensures network reliability, with LTE/4G serving as a seamless fallback during LoRaWAN network congestion or disruptions. Additionally, LTE/4G connectivity extends geographic coverage to areas where LoRaWAN may be unavailable, thereby maintaining uninterrupted operation even in challenging terrains.

**Figure 10:**
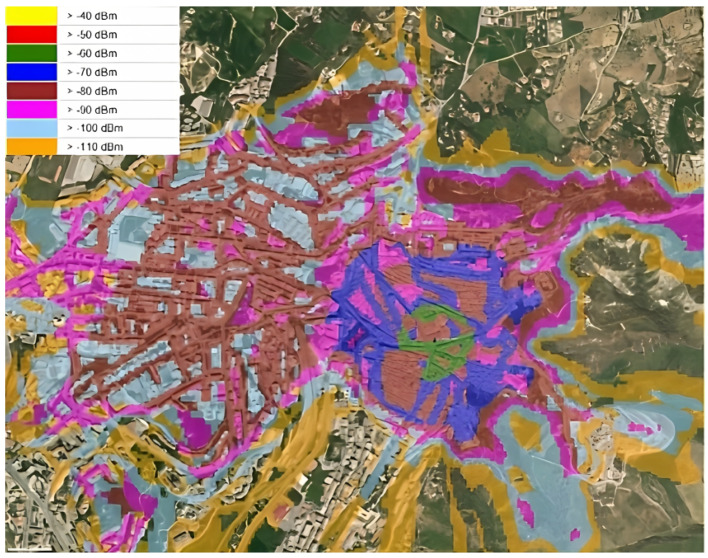
LoRaWAN coverage using a 868 MHz mobile unit with an antenna in the urban center of Caltanissetta, along with the corresponding decibel–milliwatt (dBm) legend.

Figure 10 shows the LoRaWAN coverage using a 868 MHz mobile unit with an antenna in the urban center of Caltanissetta, along with the corresponding decibel–milliwatt (dBm) legend. It results from an RF planning tool used to study the LoRaWAN coverage for different antenna configurations and placements.

Sustainability is another integral aspect of this case scenario. The ecosystem optimizes the energy consumption of its Intelligent Transport System (ITS) components, aligning with broader goals of environmental responsibility. For example, the energy absorption of traffic signal systems is carefully managed, ensuring efficient resource utilization without compromising functionality.

This example illustrates how the thoughtful integration of technologies such as LoRaWAN, LTE/4G, and advanced cloud-based platforms can transform urban environments. By enhancing connectivity, reliability, and scalability, and by embedding sustainability at its core, the Caltanissetta ecosystem serves as a blueprint for addressing contemporary urban challenges while paving the way for smarter and greener cities.

## 5. Future Perspectives

The integration of edge and cloud computing, also including the implementation of AI algorithms at the edge and/or on the cloud, presents new challenges for both fundamental and industrial research. This rapidly evolving landscape promises to reshape how we process data and deploy applications. In particular, edge computing is expected to significantly expand its impact with the growth of smart and autonomous devices in heterogeneous environments and use cases. These devices will enhance the IoT paradigm by integrating AI capabilities, enabling local data processing to reduce latency and transmit lighter and more secure data packets. This evolution will, for instance, lead to greater technological maturity in network protocols, making current LPWANs more suitable for supporting remote control actions and firmware patching. Local processing will become essential for applications requiring real-time decision-making, such as telemedicine, autonomous mobility, and industrial automation. Distributed models of federated learning and AI will allow AI training on decentralized edge devices, preserving data privacy while increasing the level of service personalization in smart city and industrial applications. At the same time, cloud computing will continue to evolve, driven by innovations such as serverless computing and the secure containerization of microservices. New serverless models will enable efficient application scaling while maintaining the underlying infrastructure’s performance, while enhanced containerization mechanisms will support seamless deployment across cloud and edge environments. Cybersecurity will remain one of the biggest challenges: the growing complexity of CPSs will require increasingly advanced security models to counter new forms of cyber threats, potentially capable of compromising systems more rapidly and on a broader scale. Technologies such as the blockchain will need to evolve to enhance security and transparency in transactions within edge–cloud ecosystems. A potential revolution lies in quantum computing, which, despite being in an early stage, is expected to integrate rapidly with edge–cloud platforms. Quantum computing promises unprecedented impact in terms of computational power. However, it will be important to reduce cost to access this technology. Therefore, it will be possible to solve complex problems and to complement traditional computing architectures for specialized tasks. In [60], the authors present and discuss an experimental implementation of a free-space quantum secure direct communication (QSDC) system based on single photons. It is an advanced technology that enables secure communication over free-space channels (e.g., atmosphere or outdoor space) without the need for a pre-shared encryption key. This technology has practical applications in various fields, including military and government communications, securing critical infrastructure and industrial plants, banking, healthcare and sensitive data protection, emergency and disaster recovery, etc. In [61], the authors surveyed the recent advances in quantum information processing, sensing, and communications, with the objective of identifying the associated knowledge gaps and formulating a roadmap for their future evolution. Finally, network infrastructure will also evolve to support efficient resource management in distributed computing environments. Mitigating latency issues and ensuring reliability are necessary to accommodate the growing complexity of CPSs.

## 6. Conclusions

In today’s rapidly evolving technology landscape, IoT, edge, and cloud systems are at the forefront of transforming the way that data are processed, shared, and used. This paper has provided an in-depth exploration of the cutting-edge technologies driving this transformation, from communication protocols such as MQTT, CoAP, and OPC UA, to the integration of advanced AI deployment tools such as Kubernetes, ONNX, and TensorFlow Lite. These innovations are setting new standards for data security, privacy, and ethical use across industries, as well as improving interoperability and scalability. Hybrid edge–cloud architectures have emerged as a foundation for modern distributed systems, necessitating robust strategies for securing containerized microservices. The convergence of long-range and low-power technologies (e.g., LoRaWAN), broadband networks and infrastructures (e.g., 5G and fiber-optic) capable of supporting intensive data collection from distributed sensor networks, and cloud-based platforms (e.g., Stack4Things) will be instrumental in addressing the complex challenges of urban environments as we move further into the smart city era. Smart cities are a key domain where IoT, AI, and cloud–edge collaboration can demonstrate their full potential. Smart cities are a key domain where IoT, AI, and cloud-edge collaboration can demonstrate their full potential. These technologies are reshaping the urban workflows of each aspect involved in this modification, from optimizing traffic flows and energy consumption to enabling the real-time monitoring of environmental conditions. Moreover, as the volume of data generated by IoT devices grows exponentially, edge computing is emerging as a critical enabler for reducing latency, improving real-time decision-making, and minimizing the strain on centralized cloud resources. This paradigm shift enhances the responsiveness of IoT systems and supports more sustainable infrastructure by optimizing resource allocation. Ethics and security remain paramount as these technologies mature. With growing concerns about data breaches and surveillance, the industry must prioritize building systems that uphold stringent privacy standards while maintaining transparency and trust. Integrating federated learning and privacy-preserving AI frameworks can be significant steps forward in this direction. Looking ahead, future research and development must focus on several key areas. First, optimizing communication protocols and AI frameworks to ensure seamless integration across diverse devices and platforms is essential. This includes fostering greater interoperability to reduce the fragmentation of IoT ecosystems. Second, advancing low-power and energy-efficient technologies will be crucial to supporting the growing number of connected devices while minimizing environmental impact. Third, more robust mechanisms for AI explainability and governance will be necessary to ensure ethical and fair decision-making in systems reliant on automated intelligence. The (ongoing) advancement in communication protocols, AI frameworks, and cloud technologies holds great promise for the next generation of connected systems. By leveraging these innovations, industries can unlock unprecedented levels of efficiency, innovation, and societal benefits. The journey from sensors to data intelligence is not merely about technological capability but also about fostering a vision where data-driven insights lead to smarter, more connected, and more equitable solutions for all. The future of IoT and AI lies in an all-encompassing collaboration among devices, systems, and human creativity, pushing the boundaries of what is possible as we enter a new era of technological integration.

## Figures and Tables

**Table 1:** Key techniques to reduce energy consumption in deep learning applications, with a focus on open-source tools and examples.

Category	Technique	Description	Open-Source Tools/Examples
Model Optimization	Pruning	Removes insignificant weights to reduce model complexity.	PyTorch Pruning, TensorFlow Model Optimization Toolkit, https://pytorch.org/ accessed on 27 February 2025
Model Optimization	Quantization	Reduces parameter precision (e.g., FP32 to INT8) for energy efficiency.	TensorFlow Lite, ONNX Runtime quantization, https://www.tensorflow.org/ accessed on 27 February 2025
Model Optimization	Knowledge Distillation	Transfers knowledge from a large model to a smaller, efficient one.	Hugging Face Transformers (DistilBERT)
Model Optimization	Specialized Architectures	Designs models optimized for fewer parameters and computations.	MobileNet, EfficientNet
Execution Optimization	Batch Normalization Folding	Combines redundant operations (e.g., BatchNorm and Convolution) to save computations.	TensorFlow Model Optimization Toolkit
Execution Optimization	Runtime Pruning	Dynamically removes unnecessary computations during execution.	TVM, OpenVINO
Data Compression	Model Compression	Compresses models to reduce memory usage.	TensorFlow Lite
Data Compression	Sparse Representations	Uses sparse data structures to save memory and computations.	PyTorch SparseTensor
Hardware Optimization	Specialized Hardware	Employs open-source hardware designs optimized for deep learning tasks.	RISC-V ML accelerators
Hardware Optimization	Dynamic Voltage and Frequency Scaling (DVFS)	Dynamically adjusts processor voltage and frequency to save energy.	Custom implementations
Resource Reduction	Edge Computing	Processes data on edge devices to minimize energy-intensive cloud communication.	TensorFlow Lite for Edge, PyTorch Mobile
Resource Reduction	Federated Learning	Implements distributed training to avoid large data transfers.	TensorFlow Federated
Smart Scheduling	Task Scheduler Optimization	Plans tasks efficiently to save energy.	Apache MXNet
Algorithmic Adjustments	Early Exit Models	Introduces early exits for simple inputs, reducing computation.	BranchyNet (open-source implementations)
Algorithmic Adjustments	Reducing Update Frequency	Reduces the frequency of weight updates during training.	Modifications to SGD algorithms

**Table 2:** Comparison of IoT Protocols: MQTT, CoAP, OPC UA, LwM2M.

Feature	MQTT	CoAP	OPC UA	LwM2M
Protocol Type	Pub/Sub (message broker)	RESTful (Client-Server)	Pub/Sub + Client–Server	Hierarchical Client–Server
Architecture	Central broker	Peer-to-Peer	Centralized and distributed	CoAP-based with hierarchical models
Efficiency	Low bandwidth consumption	Highly efficient for IoT	Suitable for complex industrial systems	Highly optimized for resource-constrained devices
OSI Layer	Application Layer (TCP/IP)	Application Layer (UDP)	Application Layer (TCP/IP, HTTP/HTTPS)	Application Layer (UDP, DTLS)
Security	TLS for encryption	DTLS for encryption	End-to-end encryption, advanced authentication	DTLS-based encryption and authentication
Scalability	High for sensors and actuators	Limited to small IoT systems	Scalable for industrial applications	Suitable for IoT networks with constrained resources
Compatibility	Suitable for general IoT	Specific for lightweight applications	Optimized for industrial systems and SCADA	Designed for embedded devices
Latency	Very low	Very low	Depends on configuration	Very low, optimized for quick responses
Use Cases	Sensors and actuators, smart home	Lightweight IoT applications	Industrial automation, IIoT	Remote management of resource-constrained IoT devices

## Abbreviations

The following abbreviations are used in this manuscript:

IoT	Internet of Things
AI	Artificial Intelligence
ML	Machine Learning
DL	Deep Learning
LoRa	Long Range
LoRaWAN	Long-Range Wide-Area Network
